# Cortical activation abnormalities in bipolar and schizophrenia patients in a combined oddball–incongruence paradigm

**DOI:** 10.1007/s00406-020-01168-1

**Published:** 2020-07-24

**Authors:** Lisa Rauer, Sarah Trost, Aleksandra Petrovic, Oliver Gruber

**Affiliations:** 1 Section for Experimental Psychopathology and Neuroimaging, Department of General Psychiatry, University Hospital Heidelberg, 69115 Heidelberg, Germany; 2 Department of Psychiatry and Psychotherapy, Center for Translational Research in Systems Neuroscience and Clinical Psychiatry, University Medical Center Göttingen, 37075 Göttingen, Germany

**Keywords:** Bipolar disorder, Schizophrenia, fMRI, Attention, Neuroimaging, Affective disorders

## Abstract

Patients with bipolar disorder and schizophrenia often suffer from severe cognitive impairment even during times of remission. This study investigated the pathomechanisms underlying their deficits in cognitive control. A combined oddball–incongruence fMRI task was applied to examine similarities and differences of neural activation patterns between patients and healthy controls. Bipolar and schizophrenia patients demonstrated hyperactivations in the intraparietal cortex during the oddball condition. Furthermore, bipolar patients revealed diagnosis-specific hyperactivation in the left middle frontal gyrus, precentral gyrus, anteroventral prefrontal cortex and orbitofrontal cortex regions compared to schizophrenia patients and healthy individuals. In comparison to healthy controls the patients showed hypoactivations in the inferior frontal junction and ventral pathway during the cognitively more demanding incongruence. Taken together, bipolar patients seem to recruit frontal and parietal areas during the oddball condition to compensate for potential deficits in their attentional network. During more challenging tasks, i.e., the incongruence condition, their compensatory mechanisms seem to collapse leading to hypoactivations in the same frontal areas as well as the ventral pathway.

## Introduction

Attention can be defined as the frail balance between concentration on task-relevant information and a continuous unconscious scanning of the environment for stimuli that demand behavioral responses (background monitoring). Selective attention processes underlie cognitive control, which is the ability to focus on a specific stimulus while other stimuli might be present. A possible way to examine cognitive control is through stimulus–response compatibility tasks such as Stroop or incongruence tasks. The Stroop task induces interference effects, so-called response conflicts, by requesting the participants to label a color word’s color, but not its meaning (e.g., the word “green” printed in red) [1]. The resulting decrease in accuracy and reaction time is called “Stroop effect” [2]. On a neurofunctional level, activation differences in anterior cingulate, prefrontal, posterior medial frontal, inferior lateral prefrontal, intraparietal and inferior parietal cortices, as well as occipito-temporal areas have been reported to be associated with cognitive control processes dealing with incongruence [3,4].

Whereas the incongruence effect is elicited by response conflicts, oddball tasks have been related to a series of cognitive processes including categorization, response selection, execution of response, etc. and do not simply map one particular course of action [5]. However, involvement of the ventral attention network in these tasks has been demonstrated multiple times. Studies involving detection of unattended, but salient and behaviorally relevant stimuli indicate an impact of functional properties of frontoparietal regions (in particular the temporoparietal junction). Both tasks require a shielding of task-relevant processing from task-irrelevant stimulus information. Incongruence and oddball tasks share activation patterns in similar frontoparietal networks proposing a similar underlying network for cognitive control processes [4].

Attentional deficits and their connection to psychiatric disorders have a long history of intense research. In particular schizophrenia has been extensively studied regarding difficulties and dysfunctions in cognition and information processing [6], for example processing speed [7], attention [8] and cognitive inhibition [9]. Furthermore, studies reported deficits in all aspects of cognitive performance in acute as well as remitted patients [10,11]. Similar results could be found in bipolar disorder [12]. It is important to note that cognitive impairment not only occurs during acute episodes but can also be present in euthymic patients. Two different meta-analytic reviews showed that neuropsychological and -cognitive function across various tasks (e.g., executive functioning, verbal learning, etc.) was impaired in euthymic bipolar patients compared to healthy controls [13,14]. Deficits in cognitive function might be influenced by following factors: prior experience of psychosis, duration of illness, frequency of manic and depressive episodes and age of onset [15,16]. Comparisons between the two disorders report comparable difficulties in executive function of bipolar patients and schizophrenia patients [17,18].

These results are further supported by functional magnetic resonance imaging (fMRI) studies, which investigated neural correlates of underlying cognitive impairments that have been observed during several neurocognitive tasks. For instance, Wolter et al*.* [19] have shown that in the same combined oddball–incongruence task, schizophrenia patients demonstrated hyperactivity in the intraparietal cortex during the oddball and incongruence task. Furthermore, there are multiple fMRI studies investigating cognitive control processes in schizophrenia or bipolar patients compared to healthy controls. However, there are almost no studies comparing between-diagnoses effects. Seok Jeong et al*.* found activation in the right precentral gyrus in schizophrenia patients but not in the healthy control group during a Stroop task [20]. Furthermore, Wagner et al*.* and Laurens et al*.* presented hypoactivations in the fronto-thalamic network as well as cingulate regions in schizophrenia patients [21,22]. These findings were confirmed by two meta-analyses [23,24]. Moreover, hypoactivation was shown consistently in bipolar patients in the right inferior frontal gyrus across various cognitive control studies including two meta-analyses [23,25–27]. Additionally, hyperactivation was found in bipolar patients compared to healthy controls in the inferior thalamus and left putamen [28]. To summarize, functional abnormalities have been demonstrated in both disorders during executive function paradigms but need further validation to elucidate potential pathomechanisms underlying cognitive control deficits.

In the present study, a combined oddball–incongruence paradigm [4,19] was employed to investigate the potential differences in cognitive control processes of bipolar disorder and schizophrenia patients. By comparing behavioral and fMRI data of both diagnoses and healthy individuals, it was aimed to gain a deeper understanding of the pathophysiology of bipolar disorder and schizophrenia.

## Method

### Participants

A total of 90 subjects—20 bipolar, 30 schizophrenia patients and 40 healthy controls—took part in this study. Patients were recruited from the Department of Psychiatry and Psychotherapy, University Medical Center Göttingen. All patients met the criteria of schizophrenia or bipolar disorder according to International Statistical Classification of Diseases and Related Health Problems 10 (ICD-10) classification standards. Both acute and partly remitted patients were involved in the study depending on their overall cognitive state. All healthy controls were recruited from the local population and matched for age, education level and gender, refer to Table 1 for further details. For bipolar patients, symptom severity was assessed using the Montgomery–Asberg Depression Rating Scale (MADRS) and the Young Mania Rating Scale (YMRS); while for schizophrenia patients, the MADRS and Positive and Negative Symptom Scale (PANSS) was used.

**Table 1:** Demographic and clinical data of patients with bipolar disorder, schizophrenia and healthy controls

	Bipolar disorder		Schizophrenia		Healthy controls	
	*N*	Mean (SD)	*N*	Mean (SD)	*N*	Mean (SD)
*Demographic data*						
Total	20	–	30	–	40	–
Gender						
Female	12	–	4	–	15	–
Male	8	–	26	–	25	–
School education						
< 9 years	2	–	3	–	1	–
10 years	5	–	6	–	3	–
> 13 years	13	–	21	–	36	–
Handedness						
Right	20	–	26	–	37	–
Left	0	–	4	–	3	–
Age						
Years	–	39.30 (12.54)	–	29.83 (7.79)	–	33.10 (9.10)
*Clinical data*						
Age of onset	–	26.25 (10.39)	–	23.29 (5.08)	–	–
MADRS	–	8.45 (7.56)	–	9.37 (7.03)	–	–
CGI	–	3.55 (1.23)	–	3.9 (1.12)	–	–
YMRS	–	3.85 (4.55)	–	–	–	–
PANSS	–	–	–	49.73 (12.15)	–	–
*Medication*						
Atypical antipsychotics	14	–	27	–	–	–
Typical antipsychotics	7	–	4	–	–	–
Antidepressants	11	–	11	–	–	–
Mood stabilizers	4	–	0	–	–	–
Anticonvulsants	15	–	0	–	–	–

*MADRS* Montgomery–Asberg Depression Rating Scale, *CGI* Clinical Global Impression Score, *YMRS* Young Mania Rating Scale, *PANSS* Positive and Negative Syndrome Scale

Exclusion criteria included lifetime diagnoses of substance dependence, substance abuse during the last month, cannabis abuse within the last two weeks, mental retardation, dementia and neurological illnesses. Secondary psychiatric lifetime comorbidities such as anxiety or personality disorders were no exclusion criteria and were present in a few patients. However, only patients treated for the primary diagnoses of schizophrenia or bipolar disorder were included in the study.

The study protocol was reviewed and approved by the local ethics committee. Informed consent was obtained from all individual participants included in the study. The study was carried out in accordance with the latest version of the Declaration of Helsinki. Participants received an expense allowance.

## Experimental protocol

Subjects were trained on the combined oddball–incongruence paradigm (described in [4, 19]) prior to being scanned and later performed the task inside a 3 T Siemens TrioTim scanner. First, all participants were instructed to categorize geometric objects by their respective shape or color via a button press as fast and as accurately as possible. The paradigm presented five experimental conditions implemented with stimuli of two different shapes (A or B) and three colors (red, blue or white). A task cue specified whether the shape or color had to be identified in the following trial. All stimuli embodied two dimensions—a relevant and an irrelevant one. The relevant dimension was the task asked for (e.g., shape); whereas the irrelevant one was to be ignored/ neglected (e.g., color). The tasks consisted of a congruent and incongruent color condition, a congruent and incongruent shape condition and the oddball condition. The stimuli were presented in a trial-by-trial manner. Task conditions were arranged pseudo-randomly, ensuring that participants were not able to predict a task switch for the subsequent trial.

Each trial started with a demonstration of the relevant task cue for 500 ms. After a 250-ms cue-stimulus interval, the stimulus was presented for 750 ms. Participants had 1000 ms to react to the stimulus (750-ms stimulus presentation + 250-ms stimulus-cue interval). During the shape task, subjects had to press the left button with their index finger for object A and the right button with their middle finger for object B—independent of their color. In the color task, participants had to press the left button with their index finger when the object was red and the right button with their middle finger when the object was blue—independent of their shape. All participants had to respond with their right hand.

**Fig. 1:**
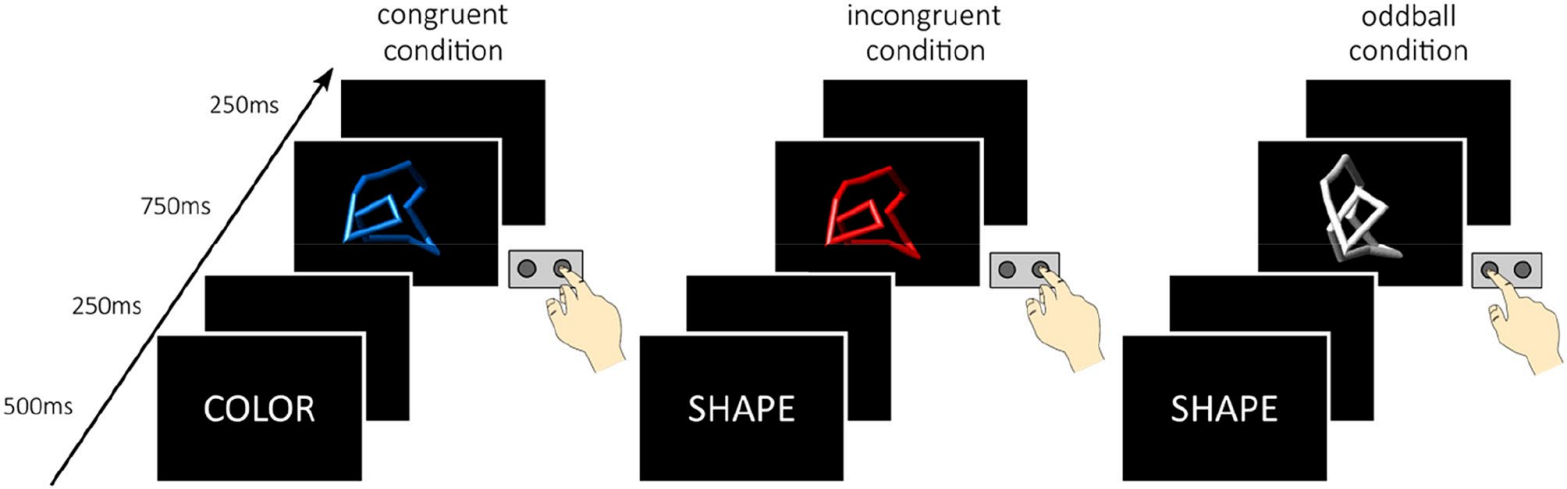
This illustration depicts an example of three of the five possible conditions. On the left, participants have to respond to the color of the object. The color and shape of the object match the same button press, which corresponds to a congruent condition. In the middle, subjects have to respond to the shape of the object. The color and shape do not match the same button press resulting in an incongruent condition. On the right, participants have to respond to the shape of the object. The presentation of a rare white object is called the oddball condition. The figure was created with Inkscape 0.92.3

Congruent stimuli were mapped on the same response button, i.e., red and object A or blue and object B. The incongruent stimuli led to a mismatch because the two stimuli dimensions were mapped on two different response buttons, (i.e., red and object B or blue and object A). In the shape task, a white object represented the so-called “oddball” stimulus, which appeared much less frequently than the congruent and incongruent stimuli. For more information see Fig. 1.

### Behavioral data analysis

Behavioral data were analyzed with IBM SPSS (SPSS Versions 25, Chicago, IL, USA). The Shapiro–Wilk test was used to check normal distribution of performance and mean reaction times of correct trials of all subjects during the oddball and incongruence tasks. Performance differences between patients and healthy controls were further analyzed with the non-parametric Kruskal–Wallis test. A one-way ANOVA was applied for the analysis of the deltas between both task conditions and mean reaction times.

### fMRI data acquisition and analyses

The experiment was performed on a 3-T MRI Scanner (Magnetom TrioTim syngo; Siemens Healthcare, Erlangen, Germany). A high-resolution T1-weighted anatomical scan (3D-MPRAGE, voxel size 1 × 1 × 1 mm^3^) was obtained for each subject. 251 functional images were acquired using a T2*-sensitive echo planar imaging (EPI) sequence (voxel size, 3 × 3 × 3 mm^3^; 20% gap; TR = 2000 ms; echo time, 30 ms; flipangle, 70°; field-of-view, 192 mm), with 33 axial slices parallel to the anterior commissure–posterior commissure plane in ascending direction.

Stimuli were presented with Presentation Software (Neurobehavioral Systems, Albany, USA) through MR-compatible LCD goggles. A button box recorded the participants’ response.

Statistical Parametric Mapping SPM8 (Wellcome Trust Centre for Neuroimaging, London, UK) was used to preprocess and analyze the functional imaging data. The EPI images were corrected for head movement by realigning them to the first image. A cutoff value of 3 mm was determined for the three planes of translation and 3° for the three planes of rotation. If this value was exceeded at any time during the paradigm, subjects were excluded from the analysis. Slice time correction was applied to the realigned and unwarped functional images. They were subsequently normalized to the MNI space and saved with a spatial resolution of 3 × 3 × 3 mm^3^. A 9-mm full-width at half-maximum (FWHM) isotropic Gaussian kernel was used for smoothing. Segmentation and co-registration of the high-resolution anatomical image was utilized with the mean EPI image. Statistical analyses applied a general linear model (GLM), which comprised five regressors (i.e., the congruent and incongruent conditions for shape and color and oddball condition). Only correct trials were included in the analyses. Linear t-contrasts were defined for measuring differential effects elicited by the experimental conditions. SPM 12 was used for the comparison of bipolar and schizophrenia patients as well as both patient groups and healthy controls during the incongruence and oddball condition. Single-subject contrast images of the oddball condition against implicit baseline and incongruence condition against implicit baseline (shape task) were taken to the second level to assess group effects. A one-way ANOVA with three groups (bipolar patients, schizophrenia patients and healthy controls) was used to determine the differences between the diagnoses as well as between the individual patient and healthy control group. Statistical effects were determined at a search criterion of *p* < 0.005, uncorrected. For brain regions with a priori hypotheses, i.e., for the left intraparietal cortex, small volume corrections (SVC) were used (left intraparietal cortex: − 36 − 60 40; coordinate taken from Gruber et al*.* [4].

## Results

### Behavioral data

First, a comparison of correct response rates and mean reaction times was conducted between bipolar and schizophrenia patients as well as healthy controls during the incongruence and oddball condition (Table 2).

**Table 2:** Behavioral performance and mean reaction time data

	Bipolar disorder		Schizophrenia		Healthy controls	
	Mean	SD	Mean	SD	Mean	SD
*Performance (%)*						
Congruence	90.96	8.44	92.05	7.21	96.01	5.03
Oddball	90.67	7.99	89.00	9.15	94.92	8.34
Incongruence	86.53	10.64	84.91	9.31	91.40	9.17
Δ Congruence vs incongruence	4.43	5.19	7.13	6.29	4.61	6.62
Δ Congruence vs oddball	0.29	6.65	3.05	7.13	1.09	5.53
*Reaction time (ms)*						
Congruence	510.44	90.38	518.60	76.14	461.38	63.52
Oddball	533.63	100.86	545.34	76.39	477.37	69.55
Incongruence	526.12	93.37	530.91	77.35	470.35	62.02
Δ Congruence vs incongruence	15.69	22.59	12.49	32.34	8.97	27.29
Δ Congruence vs oddball	23.19	24.17	26.73	32.37	15.99	27.59

*ms* milliseconds, *SD* standard deviation

Performance, defined as correct response rates, during the oddball condition was significantly affected by diagnosis (*H*(2) = 14.841, *p* = 0.001). Follow-up analyses resulted in significant differences between schizophrenia patients and healthy controls (*p* = 0.001, *r* = − 0.422) as well as bipolar patients and healthy controls (*p* = 0.017, *r* = − 0.356), but not between the patient groups (*p* = 1.000, *r* = 0.047), indicating worse performance of both patient groups compared to healthy controls. Furthermore, significant differences in the performance during the incongruent condition could be revealed (H(2) = 12.247, *p* = 0.002). Schizophrenia patients presented significantly worse performance compared to healthy controls (*p* = 0.002, *r* = − 0.408). There was no significant difference in the performance between bipolar and schizophrenia patients (*p* = 1.000, *r* = 0.136) as well as bipolar disorder and healthy individuals (*p* = 0.138, *r* = − 0.258). Performance data show the impairments of schizophrenia patients during both conditions. Bipolar patients displayed deficits compared to healthy controls during the oddball but not incongruent condition. The deltas between the congruence versus incongruence and congruence versus oddball condition in the performance data did not reveal any significant variations between patients or healthy controls (Δ congruence vs incongruence: *F*(2, 89) = 1.731, *p* = 0.183, *r* = 0.196, *ω*^2^ = 0.016; Δ congruence vs oddball: *F*(2, 89) = 1.335, *p* = 0.269, *r* = 0.173, *ω*^2^ = 0.007).

A one-way ANOVA reported significant modulation of the groups’ mean reaction time by diagnosis during the oddball condition (*F*(2, 89) = 7.155, *p* = 0.001, *r* = 0.376, *ω*^2^ = 0.120). Pairwise comparisons with adjusted *p*-values revealed significantly slower reaction times of the schizophrenia group compared to healthy individuals (*p* = 0.002). Furthermore, increased reaction times of bipolar patients compared to healthy controls reached significance (*p* = 0.031). Mean reaction times between the two patient groups did not differ. In the incongruence condition, a between-group effect could be found (*F*(2, 89) = 6.681, *p* = 0.002, *r* = 0.369, *ω*^2^ = 0.115). The processing speed of the two patient groups appeared significantly slower than in the control group (SCZ vs. HC: *p* = 0.003; BD vs HC: *p* = 0.021) and there was no significant difference between the patient groups. The difference between congruence versus incongruence as well as congruence versus oddball condition did not reveal any significant variations in the reaction times of the groups (Δ congruence vs incongruence: *F*(2, 89) = 0.399, *p* = 0.672, *r* = 0.095, *ω*^2^ = − 0.014; Δ congruence vs oddball: *F*(2, 89) = 1.273, *p* = 0.285, *r* = 0.169, *ω*^2^ = 0.006).

### fMRI results—oddball condition

**Fig. 2:**
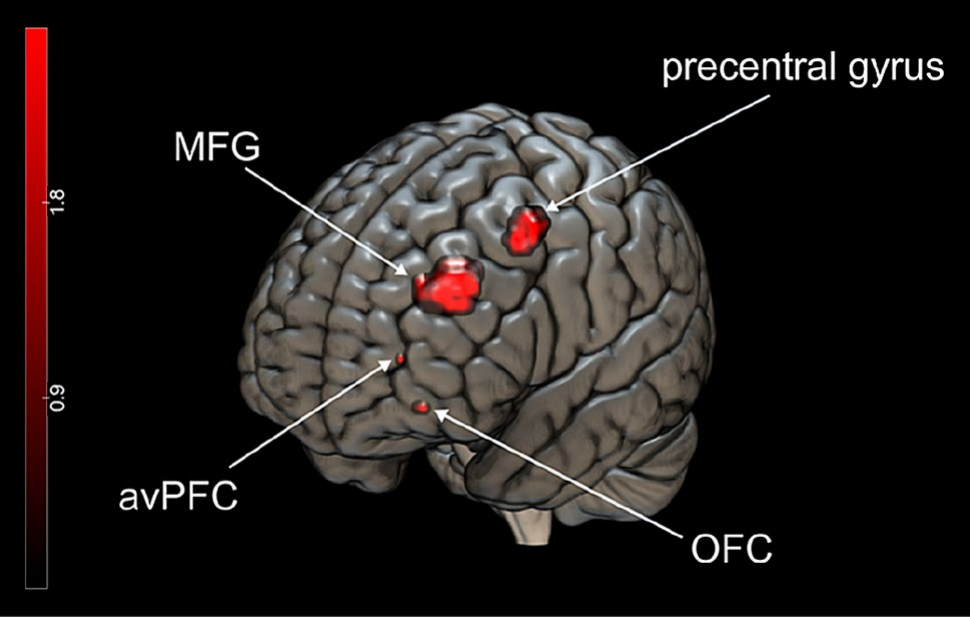
Bipolar patients compared to schizophrenia patients revealed diagnosis-specific hyperactivation evoked by the oddball condition in the left middle frontal gyrus (− 36 33 30; *p* = 0.005, uncorrected), left orbitofrontal cortex (− 24 39 − 12; *p* = 0.005, uncorrected), left precentral gyrus (− 45 6 51; *p* = 0.005, uncorrected) and left anteroventral prefrontal cortex (− 21 48 6; *p* = 0.005, uncorrected). The figure was created with MRIcroGL and Inkscape 0.92.3. *MFG* middle frontal gyrus, *OFC* orbitofrontal cortex, *avPFC* anteroventral prefrontal cortex

At first, the comparison between bipolar disorder patients and healthy controls revealed hyperactivation of the bipolar group in the left hemisphere, in regions such as the middle frontal gyrus (MFG), precentral gyrus, orbitofrontal cortex (OFC) and anteroventral prefrontal cortex (avPFC) (Table 3). All of these areas were also found to be hyperactivated in bipolar patients in comparison to schizophrenia patients (Fig. 2). These results indicate a diagnosis-specific increase of activation in frontal brain areas of bipolar disorder patients, i.e., a qualitative difference in activation patterns between bipolar disorder and schizophrenia. In addition, purely quantitative differences of activation between bipolar and schizophrenia patients, i.e., diagnosis-unspecific hyperactivations, were found in the intraparietal cortex. Bipolar patients presented enhanced activation in the intraparietal cortex bilaterally compared to healthy controls and schizophrenia patients. In addition, and in line with previous results from Wolter et al. [19], hyperactivation in the right intraparietal cortex of schizophrenia patients compared to healthy controls could be replicated.

**Table 3:** Overview of diagnosis-specific and -unspecific hyperactivations of bipolar and schizophrenia patients during the oddball condition

Region MNI coordinates (*t*-value)	BD	SCZ	HC	BD > HC	SCZ > HC	BD > SCZ
*Diagnosis-specific hyperactivations*						
L MFG	− 36 33 30 (4.04)*	n.s	[− 36 33 30 (1.82)]	[− 36 36 30 (2.58)]	n.s	− 36 33 30 (4.28)*
L precentral gyrus	− 45 0 51 (4.47)*	− 48 − 6 54 (3.06)*	− 48 − 9 54 (7.96)	− 45 9 51 (2.90)*	n.s	− 45 6 51 (3.42)*
L OFC	− 21 39 − 9 (3.88)*	n.s	n.s	− 18 39 − 12 (2.84)*	n.s	− 24 39 − 12 (2.87)*
L avPFC	− 21 39 15 (3.30)*	n.s	n.s	− 27 45 15 (2.66)*	n.s	− 21 48 6 (2.81)*
*Diagnosis-unspecific hyperactivations*						
R intraparietal cortex	33 − 54 60 (5.37)	27 − 54 48 (5.38)	27 − 54 51 (7.04)	45 − 48 57 (2.95)*	[33 − 72 57 (2.38)]	[42 − 42 66 (2.05)]
L intraparietal cortex	− 30 − 51 39 (6.57)	− 27 − 63 51 (5.38)	− 24 − 54 54 (8.16)	− 36 − 54 42 (3.69)*/ − 36 − 54 39 (3.41)**	[− 33 − 69 51 (2.36)]	− 36 − 54 39 (2.67)*/ − 36 − 51 39 (2.97)*

*L* left, *R* right, *BD* bipolar disorder patients, *HC* healthy controls, *SCZ* schizophrenia patients, *avPFC* anteroventral prefrontal cortex, *MFG* middle frontal gyrus, *OFC* orbitofrontal cortex

Activations are reported at *p* < 0.05, corrected for family-wise error rate (FWE); *activation at *p* < 0.005, uncorrected; **activation at *p* < 0.05, FWE-corrected for small volume (6 mm sphere) around a priori coordinates from Gruber et al*.* (2009); [] activation at *p* < 0.05, uncorrected

**Fig. 3:**
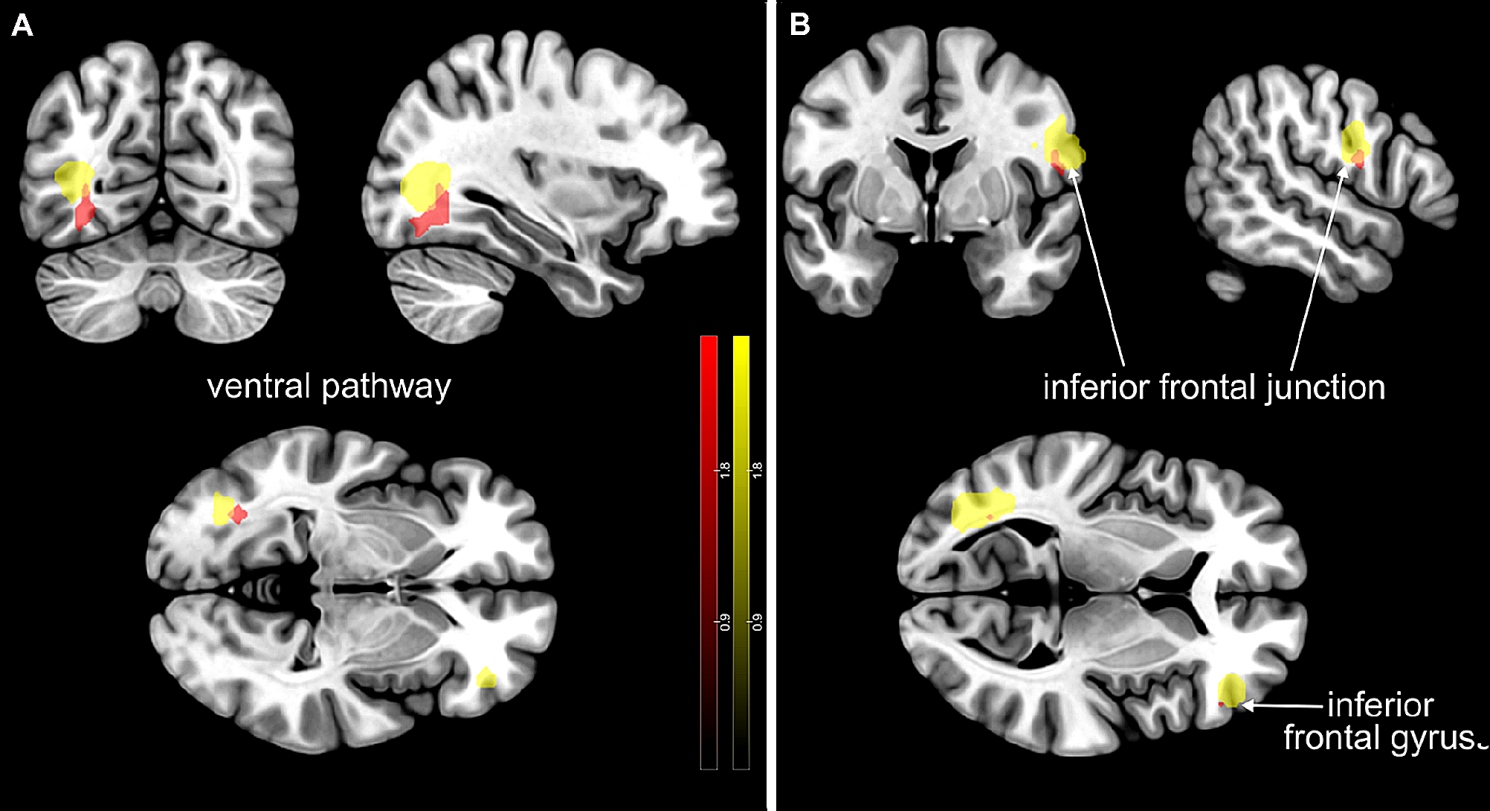
Bipolar (red) and schizophrenia (yellow) patients demonstrated diagnosis-unspecific hypoactivations in **a** the ventral pathway (BD: − 33 − 66 − 3, *p* < 0.005, uncorrected; SCZ: − 36 − 69 9, *p* < 0.005, uncorrected) and **b** the inferior frontal junction (BD: 54 0 18, *p* < 0.005, uncorrected; SCZ: 42 6 27, *p* < 0.005, uncorrected) and inferior frontal gyrus (BD: 48 36 6, *p* < 0.005, uncorrected; SCZ: 42 42 6, *p* < 0.005, uncorrected) in comparison to healthy controls. The figure was created with MRIcroGL and Inkscape 0.92.3

Among the changes of brain activity found in bipolar and schizophrenia patients, some were related to deactivations instead of activations elicited by the experimental paradigm. More specifically, the outcomes showed decreased activation in the inferior frontal junction bilaterally, left ventral pathway and right pars triangularis (Fig. 3). These hypoactivations of the patients occurred independent of their exact diagnosis, i.e., they were diagnosis-unspecific (see Table 4).

**Table 4:** Overview of diagnosis-unspecific hypoactivations of bipolar and schizophrenia patients during the oddball condition

Region MNI coordinates (*t*-value)	BD	SCZ	HC	BD < HC	SCZ < HC	BD < SCZ
*Diagnosis-unspecific hypoactivation*						
L inferior frontal junction	− 42 3 33 (4.01)*	− 54 3 42 (3.92)*	− 42 0 33 (7.17)	[− 48 − 3 30 (2.19)]	− 39 − 9 30 (3.42)*	n.s
R inferior frontal junction/ precentral gyrus	42 3 27 (3.99)*	48 3 30 (3.51)*	45 3 30 (7.06)/	54 0 18 (2.81)*	42 6 27 (3.23)*/57 0 24 (4.24)*	n.s
L posterior inferior temporal cortex/ ventral pathway	− 30 − 57 − 12 (4.24)*	− 33 − 63 − 9 (5.46)	− 33 − 51 − 18 (10.41)/ − 36 − 69 − 6 (10.71)	− 33 − 66 − 3 (3.30)*	− 36 − 69 9 (4.73)*	n.s
Pars triangularis/ orbitalis/ R inferior frontal gyrus	n.s	n.s	42 36 15 (4.61)*	48 36 6 (2.65)*	42 42 6 (3.68)*	n.s

*L* left, *R* right, *BD* bipolar disorder patients, *HC* healthy controls, *SCZ* schizophrenia patients, *OFC* orbitofrontal cortex

Activations are reported at *p* < 0.05, corrected for family-wise error rate (FWE); *activation at *p* < 0.005, uncorrected; [] activation at *p* < 0.05, uncorrected

### fMRI results—incongruence condition

Brain areas that were found to be hypoactivated in schizophrenia and bipolar patients compared to healthy controls during the oddball condition, also showed hypoactivations in the incongruence condition. These regions comprise the inferior frontal junction, ventral pathway and pars triangularis.

Furthermore, previous detected hyperactivations in the left prefrontal gyrus, intraparietal cortex, MFG and avPFC during the oddball condition of the patient groups compared to healthy controls, could not be found in the incongruence condition. In fact, patients demonstrated hypoactivations in the avPFC, MFG, intraparietal cortex and prefrontal gyrus in the incongruence condition (Table 5).

**Table 5:** Overview of diagnosis-unspecific hypoactivations of bipolar and schizophrenia patients during the incongruence condition

Region MNI coordinates (*t*-value)	BD	SCZ	HC	BD < HC	SCZ < HC	BD < SCZ
*Diagnosis-unspecific hypoactivation*						
L inferior frontal junction	[− 48 6 36 (2.29)]	− 54 6 36 (3.63)* / − 48 6 27 (2.54)*	− 42 0 33 (7.16)	− 45 − 3 30 (3.63)*	− 42 − 3 27 (3.73)*	n.s
L posterior inferior temporal cortex/ventral pathway	[− 33 − 60 − 21 (2.53)]	− 42 − 72 − 6 (5.59)	− 30 − 60 − 21 (8.44)/ − 36 − 72 − 6 (9.44)	− 24 − 60 − 21 (3.47)* /− 39 − 81 3 (3.87)* /− 33 − 78 − 9 (3.52)	− 33 − 51 − 18 (4.15)*/ − 39 − 72 6 (4.16)*	[− 33 − 66 − 3 (2.30)]
R inferior frontal junction/precentral gyrus	n.s	48 3 27 (5.65)	45 3 27 (7.73)	48 0 30 (3.53)*	[51 − 6 30 (1.93)]	48 3 24 (2.71)*
L intraparietal cortex	[− 33 − 54 45 (2.14)]	− 30 − 54 45 (5.19)	− 24 − 51 51 (8.39)	− 24 − 51 51 (3.07)*	− 24 − 48 54 (3.17)*	n.s
R intraparietal cortex/superior parietal lobe	[30 − 54 51 (1.78)]	30 − 54 48 (5.59)/ 33 − 66 54 (4.92)	27 − 51 51 (8.10)	30 − 42 45 (2.71)*/ 21 − 57 48 (2.82)*	21 − 54 63 (2.93)*	[33 − 69 57 (2.15)] / [24 − 57 51 (1.74)]
L precentral gyrus	[− 45 − 3 51 (1.87)]	− 48 − 3 54 (4.15)*	− 48 − 6 54 (8.38)	− 51 − 6 51 (2.99)*	− 48 − 9 51 (3.32)*	n.s
L MFG	[− 39 33 30 (1.69)]	− 45 30 36 (3.01)*	− 27 27 18 (5.87)	− 33 24 21 (3.10)*	[− 33 33 15 (2.52)]	[− 48 27 39 (2.04)]
L avPFC / L OFC	− 18 33 6 (3.36)*	− 21 33 − 3 (3.38)*	− 27 39 − 3 (6.69)	− 30 42 − 3 (2.87)*	[− 21 39 3 (2.22)]	n.s
Pars triangularis/orbitalis/R inferior frontal gyrus	n.s	n.s	27 39 0 (4.77)	45 36 6 (4.38)*	36 42 0 (2.69)*	n.s

*L* left, *R* right, *BD* bipolar disorder patients, *HC* healthy controls, *SCZ* schizophrenia patients, *avPFC* anteroventral prefrontal cortex, *IFS* inferior frontal sulcus, *MFG* middle frontal gyrus

Activations are reported at *p* < 0.05, corrected for family-wise error rate (FWE); *activation at *p* < 0.005, uncorrected; [] activation at *p* < 0.05, uncorrected

## Discussion

This fMRI study aimed to shed light on pathophysiological similarities and differences of neural correlates of cognitive control processes in bipolar related to schizophrenia patients and healthy controls by examining an oddball and incongruence task. It was of special interest to examine: (i) diagnosis-specific and (ii) diagnosis-overlapping cognitive processing effects. Both, response conflict (incongruence condition) and contextual mismatch (oddball condition), induced activation in superior frontal and parietal, central and occipital regions that have been shown to be associated with cognitive control processes [29–34]. In the current study, diagnosis-specific hyperactivation of the bipolar disorder group in comparison to healthy controls and schizophrenia patients could be found in frontal regions such as the left avPFC, OFC, precentral gyrus and MFG during the oddball condition. Increased activations in those brain areas were all specific to the bipolar patients and, hence, represent a qualitative difference in relation to schizophrenia. Enhanced activation in the intraparietal cortex, on the other hand, could be found not only in the bipolar, but also in the schizophrenia group. In addition, significant hypoactivations in both patient groups were found in frontal areas and in the ventral pathway. Results of the incongruence task demonstrated hypoactivations of schizophrenia and bipolar patients compared to healthy individuals in the ventral pathway and inferior frontal junction. However, in contrast to the oddball condition, patients revealed hypoactivations in the intraparietal cortex, MFG, avPFC and left precentral gyrus during the incongruence condition.

Hyperactivations found in frontal areas during the oddball condition were specific for the bipolar disorder group. Additionally, the behavioral data results revealed a significantly reduced accuracy of bipolar patients compared to healthy controls. Together this might indicate a malfunction of bipolar patients in cognitive control processing leading to a compensatory increase in frontal brain activation. Previous studies examining cognitive control have already shown increased activation in frontal areas of bipolar disorder compared to healthy controls [35,36]. These results suggest overall disturbed attention mechanisms in bipolar patients, resulting in potential hyperactivations of selective brain regions to maintain their performance. Additionally, it has been shown that patients with obsessive–compulsive disorder exhibited enhanced frontal activation compensating for their impaired performance on a working memory task [37].

Whereas bipolar patients recruited additional frontal areas such as the avPFC or OFC to counterbalance their deficits, schizophrenia patients might not be able to compensate for their reduced attentional capacity through hyperactivation of frontal regions in the oddball condition. Similar effects have already been shown in a study by McIntosh et al*.*, who reported increased activation of bipolar patients compared to schizophrenia patients in the OFC during the Hayling Sentence Completion test [38]. Additionally, the middle frontal gyrus has already been reported to show decreased activation in ultra-high-risk, early and chronic schizophrenia patients during a visual oddball task [39]. The authors proposed that decreased activation in the middle frontal gyrus during the oddball condition might result from disrupted processing of and response to task-relevant stimuli due to the inability of consequently neglecting task-irrelevant inputs. While dopamine regulates contextually meaningful salience in healthy controls, a dysfunctional and uncontrolled dopamine release in schizophrenia might induce inappropriate salience to irrelevant inputs.

Furthermore, it has repeatedly been shown that schizophrenia patients suffer from greater cognitive impairment than bipolar disorder patients [40–44]. Consequently, their compensation mechanisms seem to be more limited than in affective disorders which may explain the missing compensatory activation in frontal brain areas observed in this study. This explanation might also be valid for the effects exhibited in the intraparietal cortex. Wolter et al*.* [19] reported hyperactivation of schizophrenia patients in comparison to healthy controls in the intraparietal cortex applying the same paradigm. In the current study, subjects with bipolar disorder displayed significant hyperactivation in the intraparietal cortex compared to healthy controls and schizophrenia patients, whereas subjects with schizophrenia showed low-threshold activation differences in comparison to healthy controls. It seems that bipolar and schizophrenia patients used similar compensation strategies, but significantly differed in the quantitative extent. The difference to the results of Wolter et al*.* [19] could result from the increased sample size of schizophrenia patients. Individual differences in the performance capacity of schizophrenia patients influence the outcome of the group comparison. Whereas the previous study detected hyperactivation in the oddball and incongruence condition of schizophrenia patients compared to healthy individuals, it seems as if more subjects of the present sample had deficits in response conflict processing. In consequence, these deficits might result in hypoactivation of the intraparietal cortex during the incongruence condition. It has repeatedly been shown that schizophrenia comprises a very heterogeneous group of symptoms or even level of cognitive performance [45]. These differences might potentially also affect the functional outcome quantitatively. Consequently, due to a potential shift of the overall performance during the oddball condition, the effects stated by Wolter et al*.* [19], could only be replicated in a low-threshold manner.

Taken together, the compensatory mechanisms of the bipolar patients led to hyperactivations in intraparietal as well as frontal regions in the oddball condition, whereas schizophrenia patients only exhibited hyperactivation in the intraparietal cortex. The results of Melcher et al*.* [40] concur with this outcome as they found in a study with similar versions of this neuropsychological task that overall, schizophrenia patients seem to have greater dysfunctions than bipolar patients in inhibitory control and attention maintenance. They described an increased conflict and oddball effect on the reaction times of schizophrenia patients in comparison to bipolar and healthy individuals. In this study, the diagnoses did not induce a conflict and oddball effect on the reaction times or accuracy. At the descriptive level, however, schizophrenia patients demonstrated a significant difference in performance scores between congruence and incongruence as well as congruence versus oddball condition indicating deficits in the processing of incongruence and oddball effects. Therefore, bipolar patients seem to be able to compensate for their behavioral deficits, whereas schizophrenia patients have limited compensation capacities resulting in less activation of frontal areas.

Hypoactivations of bipolar and schizophrenia patients were found in the ventral pathway. Associations between the visual cortex and the temporal lobe, or more specifically its respective connections to limbic and frontal areas, make it possible to process visual information. In particular, attention to and processing of feature dimensions (such as shape or color) has been reported to activate the ventral stream [46–48]. It can be hypothesized that healthy controls might process visual stimuli more efficiently. Patients, on the other hand, might have greater deficits in directing their attention to visual stimuli to the same extent as healthy controls. Furthermore, it might be possible that auditory distraction due to scanner noises caused interference with their stimuli processing leading to attentional deficits. The current literature presents a mixed picture regarding this hypothesis, i.e., not only hypoactivation of schizophrenia patients in the ventral stream during visual perception [49–52], but also hyperactivation in the lateral occipital complex in a task involving object recognition and mapping of visual-spatial attention [53]. There seem to be no studies examining visual perception of bipolar patients with fMRI. However, neurocognitive object perception tasks demonstrated deficits in bipolar patients [54,55]. It can be concluded that the specific neural mechanisms have not yet been fully elucidated, however, object processing seems to be aberrant in bipolar disorder and schizophrenia.

In addition, diagnosis-unspecific hypoactivations of the patient groups in the inferior frontal junction might represent difficulties in shifting attention from novel to the relevant stimulus dimension. Healthy controls activate the inferior frontal junction during goal-directed and stimulus-driven attention [56,57],whereas, bipolar and schizophrenia patients exhibit impaired ability in shifting attention to the target stimulus [40,58,59].

The second task in this experiment, i.e., the incongruence condition, focused on activations in the brain responsible for response conflict and executive control. Previous studies demonstrated activation in similar brain regions, i.e., the frontoparietal network during incongruence and oddball conditions [19,31,60]. Therefore, it is no surprise that the activation patterns of healthy controls were constant across both conditions, i.e., comparable regions were activated to a similar extent. Comparable results have already been demonstrated in numerous studies, repeatedly reporting activation of the inferior frontal junction, precentral gyrus, ventral pathway, intraparietal cortex and occipital regions during oddball and incongruence tasks [32,33,61–65]. These results suggest the involvement of partly overlapping brain regions in the processing of oddball effects as well as response conflict during incongruence tasks.

Results from the comparison between the two patient groups and healthy controls in the incongruence condition revealed hypoactivations of the patient groups in areas such as the inferior frontal junction, avPFC, MFG, intraparietal cortex and superior frontal gyrus. Thereby, overlapping hypoactivations in regions such as the inferior frontal junction, ventral pathway and right pars triangularis can be observed in both conditions. These results indicate a partly shared pathophysiological decrease in activation in bipolar and schizophrenia patients during the processing of the oddball and incongruence stimuli.

However, there seem to be additionally diagnosis-specific differences in the functional responses to the oddball and incongruence stimuli. This effect might result from a variance in difficulty of the two conditions. Whereas the incongruence condition seems to be more difficult due to its stimulus–response mapping dimension, the oddball condition does not entail this dimension. As a result, the oddball task seems to be easier, which is also supported by the fact that the correct response rates in the healthy control group were significantly greater compared to the incongruence condition. In consequence, bipolar patients might exhibit more deficits in the incongruence condition than in the oddball condition resulting in hypoactivation of the left prefrontal gyrus and intraparietal cortex compared to healthy controls. A possible explanation for this effect results from a model described multiple times in the last decade [66–69]. It states that in general, the neurophysiological response curve of each individual increases with enhanced task difficulty. After exceeding an individual tipping point, the functional response decreases with further difficulty resulting in an inverted U-shaped curve. As psychiatric patients have performance deficits, their neurofunctional response increases and decreases earlier, i.e., already in easier tasks, leading to a left-shifted U-shaped curve. This means, more precisely, that during easier tasks (in this case the oddball condition) patients overcompensate with stronger activations in various brain regions. However, as soon as a certain/individual attention capacity is exceeded (i.e., the incongruence condition), they demonstrate hypoactivations as compared to healthy individuals. Following this hypothesis, it is possible that bipolar patients had difficulties with both conditions but were able to recruit frontal and parietal brain regions to support task accomplishment in the oddball condition. The incongruence condition, however, might require too much cognitive capacity so that they fail to involve assisting networks and respond with decreased activation. In the schizophrenia group, hyperactivation was found in the intraparietal cortex in the oddball condition, but shifted to hypoactivation in the incongruence condition. In consequence, it can be hypothesized that this group has greater deficits per se but struggled even more in the incongruence condition.

It is important to note that the two patient groups varied in the distribution of females and males, i.e., the bipolar group included 12 females and 8 males; whereas the schizophrenia group consisted of 4 females and 26 males. As a consequence, the analyses was repeated with gender as a covariate. Overall, the results remained consistent suggesting that the gender of the subjects did not influence the outcomes of this study. However, it has to be mentioned that the literature provides inconsistent results regarding the influence of gender on cognitive control processes. Some studies presented activation differences in the frontoparietal network during cognitive control tasks between women and men [70,71], whereas other authors ruled out sex differences in functional or resting-state connectivity networks related to cognitive control processes [72,73]. One potential interpretation of sex differences is suggested to be the influence of estradiol on cognitive control processes [74]. It can be concluded that effects of gender or sex hormones have not been fully resolved, yet. In the current study, gender does not seem to be a driving force between differences found in cognitive control processing of bipolar and schizophrenia patients as well as healthy controls.

Taken together, this study provided new insights into the functional responses underlying attentional processes induced by oddball and incongruence stimuli in bipolar and schizophrenia patients. The current results revealed the involvement of shared brain areas in the processing of oddball and incongruence stimuli, yet pathophysiological differences between bipolar and schizophrenia patients as well as healthy controls. Bipolar patients presented greater activation in frontal as well parietal brain regions during the oddball condition compared to schizophrenia and healthy individuals. Schizophrenia patients revealed low-threshold hyperactivation in the right intraparietal cortex compared to healthy controls. Additionally, both patient groups exhibited hypoactivations in the ventral pathway and frontal areas compared to healthy controls. The incongruence condition seemed to require greater cognitive capacities resulting in a collapse of compensatory mechanisms and hence hypoactivations of both patient groups compared to healthy controls in frontal and intraparietal regions. As a result, the activation levels of both psychiatric disorders seem to greatly depend on the task difficulty. However, additional studies with greater or evenly distributed sample sizes will be necessary to avoid group size effects. As it was not controlled for medication, psychopharmacological effects on brain activation cannot be excluded and might have led to variance in the results. Prospective studies might, therefore, need to solely examine medication-naïve patients to prevent psychopharmacological effects. All in all, both psychiatric disorders presented activation differences during the processing of oddball and incongruence effects compared to healthy controls. Bipolar patients seem to accomplish intermediate performance by recruiting frontal and parietal brain regions. However, as soon as the task difficulty exceeded their cognitive performance spectrum, their compensation strategies collapsed resulting in hypoactivations of corresponding brain areas.
