# Correction to: Cortical activation abnormalities in bipolar and schizophrenia patients in a combined oddball–incongruence paradigm

**DOI:** 10.1007/s00406-021-01276-6

**Published:** 2021-06-09

**Authors:** Lisa Rauer, Sarah Trost, Aleksandra Petrovic, Oliver Gruber

**Affiliations:** 1 Section for Experimental Psychopathology and Neuroimaging, Department of General Psychiatry, University Hospital Heidelberg, 69115 Heidelberg, Germany; 2 Department of Psychiatry and Psychotherapy, Center for Translational Research in Systems Neuroscience and Clinical Psychiatry, University Medical Center Göttingen, 37075 Göttingen, Germany

## Correction to: European Archives of Psychiatry and Clinical Neuroscience 10.1007/s00406-020-01168-1

The article “Cortical activation abnormalities in bipolar and schizophrenia patients in a combined oddball–incongruence paradigm” written by Lisa Rauer, Sarah Trost, Aleksandra Petrovic and Oliver Gruber was originally published electronically on the publisher’s internet portal on July 24, 2020 without open access. With the author(s)’ decision to opt for Open Choice the copyright of the article changed to © The Author(s) 2021 and the article is forthwith distributed under a Creative Commons Attribution 4.0 International License, which permits use, sharing, adaptation, distribution and reproduction in any medium or format, as long as you give appropriate credit to the original author(s) and the source, provide a link to the Creative Commons licence, and indicate if changes were made. The images or other third-party material in this article are included in the article’s Creative Commons licence, unless indicated otherwise in a credit line to the material. If material is not included in the article’s Creative Commons licence and your intended use is not permitted by statutory regulation or exceeds the permitted use, you will need to obtain permission directly from the copyright holder. To view a copy of this licence, visit http://creativecommons.org/licenses/by/4.0/. Open Access funding enabled and organized by Projekt DEAL.

